# Tofacitinib facilitates the expansion of myeloid-derived suppressor cells and ameliorates interstitial lung disease in SKG mice

**DOI:** 10.1186/s13075-019-1963-2

**Published:** 2019-08-06

**Authors:** Sho Sendo, Jun Saegusa, Hirotaka Yamada, Keisuke Nishimura, Akio Morinobu

**Affiliations:** 1 Department of Internal Medicine, Division of Rheumatology and Clinical Immunology, Kobe University Graduate School of Medicine, 7-5-2 Kusunoki-cho, Chuo-ku, Kobe, 650-0017 Japan; 2 Department of Endocrinology and Rheumatology, Kurashiki Central Hospital, 1-1-1 Miwa, Kurashiki, Okayama 710-8602 Japan

**Keywords:** Rheumatoid arthritis, Interstitial lung disease, Tofacitinib, Myeloid-derived suppressor cells, Th17 cells, Dendritic cells

## Abstract

**Background:**

Rheumatoid arthritis-associated interstitial lung disease (RA-ILD) is a sometimes life-threatening complication in RA patients. SKG mice develop not only arthritis but also an ILD resembling RA-ILD. We previously reported that tofacitinib, a JAK inhibitor, facilitates the expansion of myeloid-derived suppressor cells (MDSCs) and ameliorates arthritis in SKG mice. The aim of this study was to elucidate the effect of tofacitinib on the ILD in SKG mice.

**Methods:**

We assessed the effect of tofacitinib on the zymosan (Zym)-induced ILD in SKG mice histologically and examined the cells infiltrating the lung by flow cytometry. The effects of lung MDSCs on T cell proliferation and Th17 cell differentiation were assessed in vitro. We also evaluated the effects of tofacitinib on MDSCs and dendritic cells in vitro.

**Results:**

Tofacitinib significantly suppressed the progression of ILD compared to the control SKG mice. The MDSCs were increased, while Th17 cells, group 1 innate lymphoid cells (ILC1s), and GM-CSF+ILCs were decreased in the lungs of tofacitinib-treated mice. MDSCs isolated from the inflamed lungs suppressed T cell proliferation and Th17 cell differentiation in vitro. Tofacitinib promoted MDSC expansion and suppressed bone marrow-derived dendritic cell (BMDC) differentiation in vitro.

**Conclusion:**

Tofacitinib facilitates the expansion of MDSCs in the lung and ameliorates ILD in SKG mice.

**Electronic supplementary material:**

The online version of this article (10.1186/s13075-019-1963-2) contains supplementary material, which is available to authorized users.

## Background

Interstitial lung disease (ILD) is sometimes a complication of rheumatoid arthritis (RA), in which case it is called rheumatoid arthritis-associated ILD (RA-ILD).

Although some patients with RA-ILD respond to corticosteroid therapy [1], others do not. Corticosteroid therapies also have many side effects, and treatments with fewer side effects are desired.

Tofacitinib, a Janus kinase (JAK) inhibitor, is approved for RA treatment in humans. Although tofacitinib relieves arthritis, its effect on RA-ILD has not been elucidated. Tofacitinib suppresses the differentiation of human T cells and dendritic cells in vitro [2]. Kurasawa et al. recently reported that tofacitinib may control refractory ILD in anti-melanoma differentiation-associated 5 gene (MDA5) antibody-positive dermatomyositis [3]. These findings suggested that tofacitinib might also inhibit the development of RA-ILD.

Monocyte-derived suppressor cells (MDSCs) are immature myeloid cells with a suppressive function. MDSCs negatively control inflammation in inflamed organs such as the lungs and joints by suppressing T cells and myeloid cells [4, 5]. SKG mice, an animal model of rheumatoid arthritis, can develop not only arthritis but also ILD (SKG-ILD) [6, 7]. GM-CSF is critical for the development of ILD in SKG mice [8], but it also promotes MDSC expansion. We previously reported that the MDSCs and CD11b^+^Gr1^dim^ tolerogenic dendritic cell-like cells (CD11b^+^Gr1^dim^ tolDC-like cells) were increased in the inflamed lungs of SKG mice [9]. Although we demonstrated that the CD11b^+^Gr1^dim^ tolDC-like cells were differentiated from monocytic MDSCs in the severely inflamed lungs, and suppressed the progression of ILD, the function of MDSCs in the lung was not elucidated. We also showed that tofacitinib facilitated the expansion of MDSCs in the spleen and bone marrow (BM) and ameliorated the arthritis in SKG mice [10]. Combining these results, we hypothesized that tofacitinib ameliorates SKG-ILD by facilitating the expansion of MDSCs.

In this study, we found that tofacitinib suppressed the progression of SKG-ILD. Tofacitinib significantly increased the MDSCs and suppressed Th17 cells, group 1 innate lymphoid cells (ILC1s), and GM-CSF^+^ ILCs in the inflamed lungs. Tofacitinib also facilitated MDSC expansion in vitro. The isolated MDSCs from inflamed lungs suppressed T cell proliferation and Th17 cell differentiation ex vivo. These results collectively indicated that tofacitinib is a potential therapeutic option for RA-ILD.

## Methods

### Mice

Male SKG mice (CLEA Japan) were used at 3–20 weeks of age. All mice were kept in specific pathogen-free conditions at the Institute of Laboratory Animals, Graduate School of Medicine, Kobe University. All mice were handled in accordance with the guidelines for animal care approved by the Animal Experimentation Committee of Kobe University.

### Reagents and antibodies

Zymosan (Zym) was purchased from Alfa Aesar, 2-mercaptoethanol (2-ME) from Sigma-Aldrich, tofacitinib from Selleck Chemicals, DMSO from TOCRIS Bioscience, carboxyfluorescein diacetate succinimidyl ester (CFSE-DA) Cell Proliferation Kit from Invitrogen, RPMI 1640 Medium and Collagenase Type1 from Wako Pure Chemical Industries, fetal bovine serum (FBS) from MP Biomedicals, 1% penicillin-streptomycin from Lonza Walkersville, GM-CSF from Pepro Tech, and recombinant mouse interleukin-4 (IL-4) protein from R & D Systems. BD Phosflow Fix Buffer 1, FITC-conjugated anti-mouse Gr1 (FITC-anti-Gr1), PE-anti-mouse Gr1 (Ly6G and Ly6C), PerCP-anti-mouse CD4, PE-anti-mouse STAT1 (pY701), PE-anti-mouse STAT3 (pY705), Pacific Blue-anti-mouse STAT5 (pY694), FITC-rat IgG2b k isotype, PE-mouse IgG2a k isotype, PerCP-rat IgG2a k isotype, Pacific Blue-mouse IgG1 k isotype, purified hamster anti-mouse CD3e, purified hamster anti-mouse CD28, and purified rat anti-mouse CD16/CD32 (Fc block) were obtained from BD Biosciences. Brefeldin A, APC-anti-mouse CD11b, PE-anti-mouse F4/80, FITC-anti-mouse CD4, APC-anti-mouse CD4, PE-Cyanine7-anti-mouse CD90.2 (Thy1.2), PE-anti-mouse GM-CSF, PE-anti-mouse FoxP3, PE-anti-mouse RORγt, PE-anti-mouse T-bet, APC-anti-mouse RORγt, PE-rat IgG1 k isotype, APC-mouse IgG2a k isotype, and PE-Cyanine7-rat IgG2a k isotype were obtained from eBioscience. Cell-staining buffer, nuclear-factor fixation and permeabilization buffer, PerCP-anti-mouse/human CD11b, FITC-anti-mouse lineage cocktail (CD3/Gr1/CD11b/CD45R(B220)/Ter-119), APC-anti-mouse GATA3, PerCP-rat IgG2b k isotype, and FITC-anti-hamster IgG/rat IgG2b/IgG2a isotype were obtained from BioLegend.

### Induction of ILD in SKG mice

Male mice aged 8–9 weeks were given i.p. injections (5 mg) of Zym (Alfa Aesar) suspended in 0.5 mg normal saline as previously described [11].

### Lung histology and scoring

The left lung was inflated with 4% paraformaldehyde, embedded in paraffin, and sectioned by a cryostat. The sections were stained with hematoxylin and eosin (H&E). All images were captured using a Keyence BZ-X710, and the images were combined to obtain the entire lung shape. The percentage of the affected area was calculated by ImageJ software (Wayne Rasband), and the histological score was defined as previously described [11].

### Collection of bone marrow-, spleen-, and lung-infiltrating cells

Mice were euthanized with a high dose of pentobarbital sodium. The lung-infiltrating cells were collected from the right lungs as previously described [11]. BM and spleen cells were directly filtered through a 100-μm nylon filter and collected.

### Isolation of lung MDSCs and spleen CD4^+^ T cells

A BD FACSAria III (BD Biosciences) was used to sort MDSCs (CD11b^+^Gr1^+^cells) from the lung. CD4^+^ T cells were isolated from the spleen by MACS, according to the manufacturer’s protocol.

### Cell staining and flow cytometry

For surface staining, single-cell suspensions of lung cells, BM cells, and splenocytes were washed with cell-staining buffer (BioLegend) and stained with anti-Gr1, anti-CD11b, anti-CD11c, anti-F4/80, anti-CD4, anti-CD90.2, and anti-lineage cocktail antibodies for 30 min at 4 °C. For intracellular cytokine and nuclear factor staining, lung cells were stimulated with PMA and ionomycin for 6 h and brefeldin A for the last 4 h, as previously described [12]. The cells were stained with anti-GM-CSF, anti-T-bet, anti-GATA3, anti-RORγt, and anti-FoxP3 antibodies according to the manufacturer’s protocol. For phosphorylated STAT staining, lung cells were stained with anti-pSTAT1, anti-pSTAT3, and anti-pSTAT5 antibodies according to the manufacturer’s protocol. Flow cytometry data were acquired on a FACSCalibur or FACSVerse (BD Biosciences) and analyzed with FlowJo software (Tree Star).

### Generation of dendritic cells (DCs) from BM cells and lung-infiltrating cells in vitro

The total BM cells (1 × 10^6^) and total lung-infiltrating cells (1 × 10^6^) from naïve SKG mice were cultured in RPMI 1640 medium supplemented with 10% FBS, 1% penicillin/streptomycin, 50 μM 2-ME, and 100 ng/ml GM-CSF, with 2.5 ng/ml rIL-4, as previously described [13]. These cultures were maintained at 37 °C in 48-well plates in a 5% CO_2_-humidified atmosphere. On day 3 of culture, the supernatant was gently removed, and the medium was replaced with fresh medium containing GM-CSF with rIL-4. The cells were collected on day 5 and analyzed by flow cytometry.

### T cell proliferation and differentiation assay

Isolated spleen CD4^+^ T cells from 3-week-old SKG were incubated with 10 μM CFSE-DA according to the manufacturer’s protocol. CFSE-labeled CD4^+^ T cells (2 × 10^5^) in 200 μl of medium were cultured at 37 °C (5% CO_2_) for 3 days in a 96-well flat-bottomed plate pre-coated with 10 μg/ml anti-CD3 antibody and 5 μg/ml anti-CD28 antibody with or without the indicated proportion of lung MDSCs (e.g., 2 × 10^3^, 2 × 10^4^, or 2 × 10^5^) isolated from Zym-injected SKG mice. The CD4^+^ T cell proliferation was determined by measuring the CFSE fluorescence using flow cytometry. For the Th17 cell differentiation assay, in addition to the proliferation conditions, the following cytokines and antibodies were added: 10 ng/ml IL-6, 0.5 ng/ml TGF-β, 2.5 μg/ml anti-IFNγ, and 2.5 μg/ml anti-IL-4. CD4^+^ T cells were cultured in the Th17 cell differentiation condition with or without lung MDSCs (2 × 10^5^).

### Tofacitinib treatment

Male mice aged 8–9 weeks were given i.p. injections (5 mg) of Zym suspended in 0.5 mg normal saline as previously described [9]. Tofacitinib was dissolved in DMSO. Four weeks after the injection of Zym, tofacitinib (20 mg/kg) or DMSO was injected i.p. three times per week for 8 weeks as previously described [14]. The mice were sacrificed on day 91.

### Statistical analysis

Results are expressed as the mean ± SEM. All analyses were performed using GraphPad Prism 5 software (GraphPad). Statistical comparisons were performed using Student’s *t* test in cases with a normal distribution or the Mann-Whitney *U* test in cases with a non-normal distribution. Pearson’s or Spearman’s correlation coefficient was calculated for the correlations performed. *P* values < 0.05 were considered to be statistically significant.

## Results

### Tofacitinib treatment suppresses the ILD progression in SKG mice

**Fig. 1:**
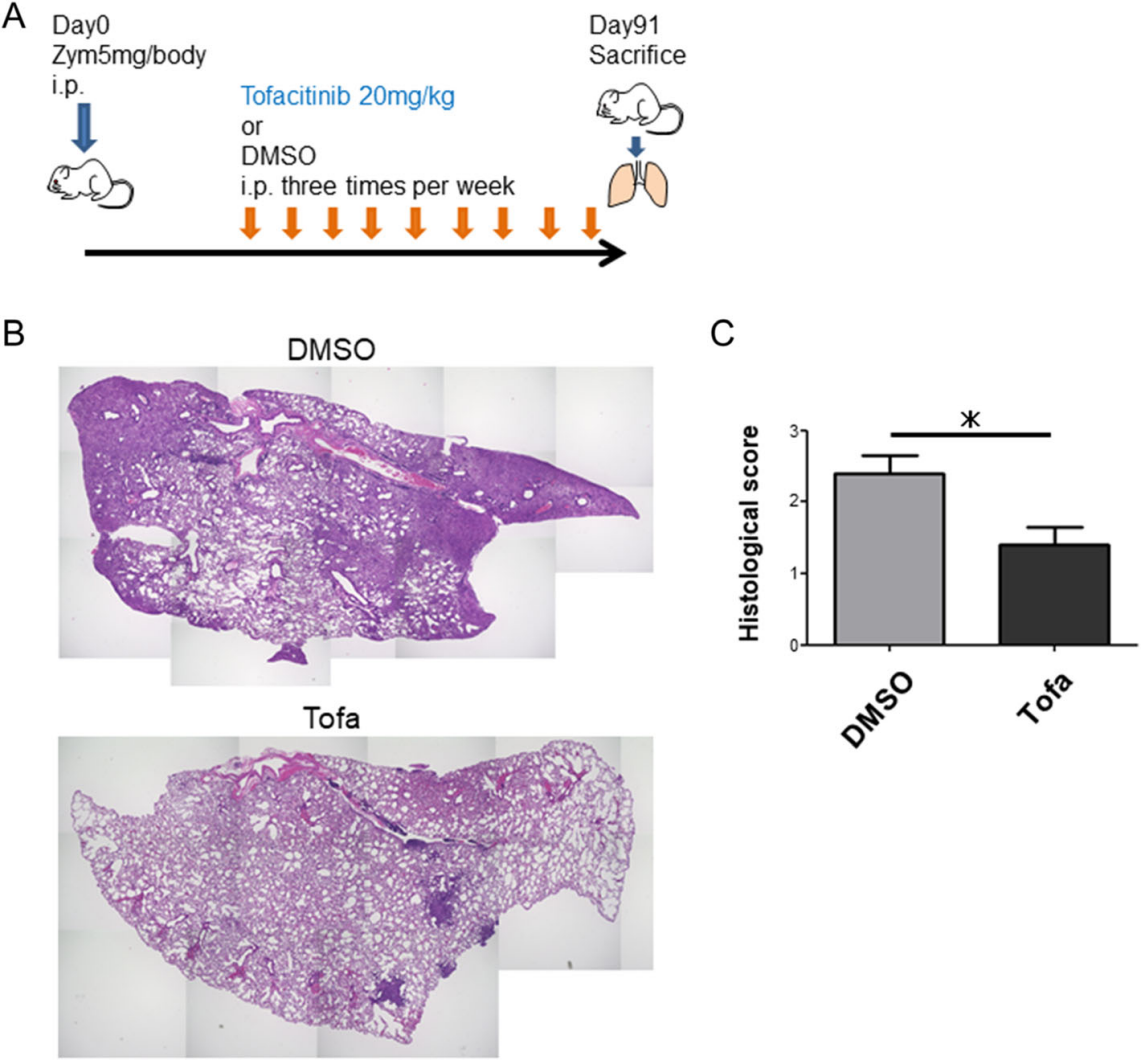
Tofacitinib ameliorates ILD in SKG mice. **a** Tofacitinib treatment protocol. Four weeks after the injection of Zym, tofacitinib (20 mg/kg) (*n* = 5) or DMSO as a control (*n* = 5) was injected i.p. three times per week for 8 weeks. The mice were sacrificed on day 91. **b** Representative histologic results from the lungs of control mice and of tofacitinib-treated mice. **c** Histological score of the lungs from control and tofacitinib-treated mice. Data are shown as the mean ± SEM. *P < 0.05, Mann-Whitney *U* tests

We first investigated the effect of tofacitinib on the progression of ILD in SKG mice (Fig. 1a). The DMSO-treated mice developed moderate or severe ILD 3 months after Zym injection (Fig. 1b). Two months of tofacitinib treatment significantly suppressed the progression of ILD compared to DMSO treatment (Fig. 1b, c).

### Tofacitinib suppresses Th17 cells, ILC1s, and GM-CSF^+^ ILCs in the lung

**Fig. 2:**
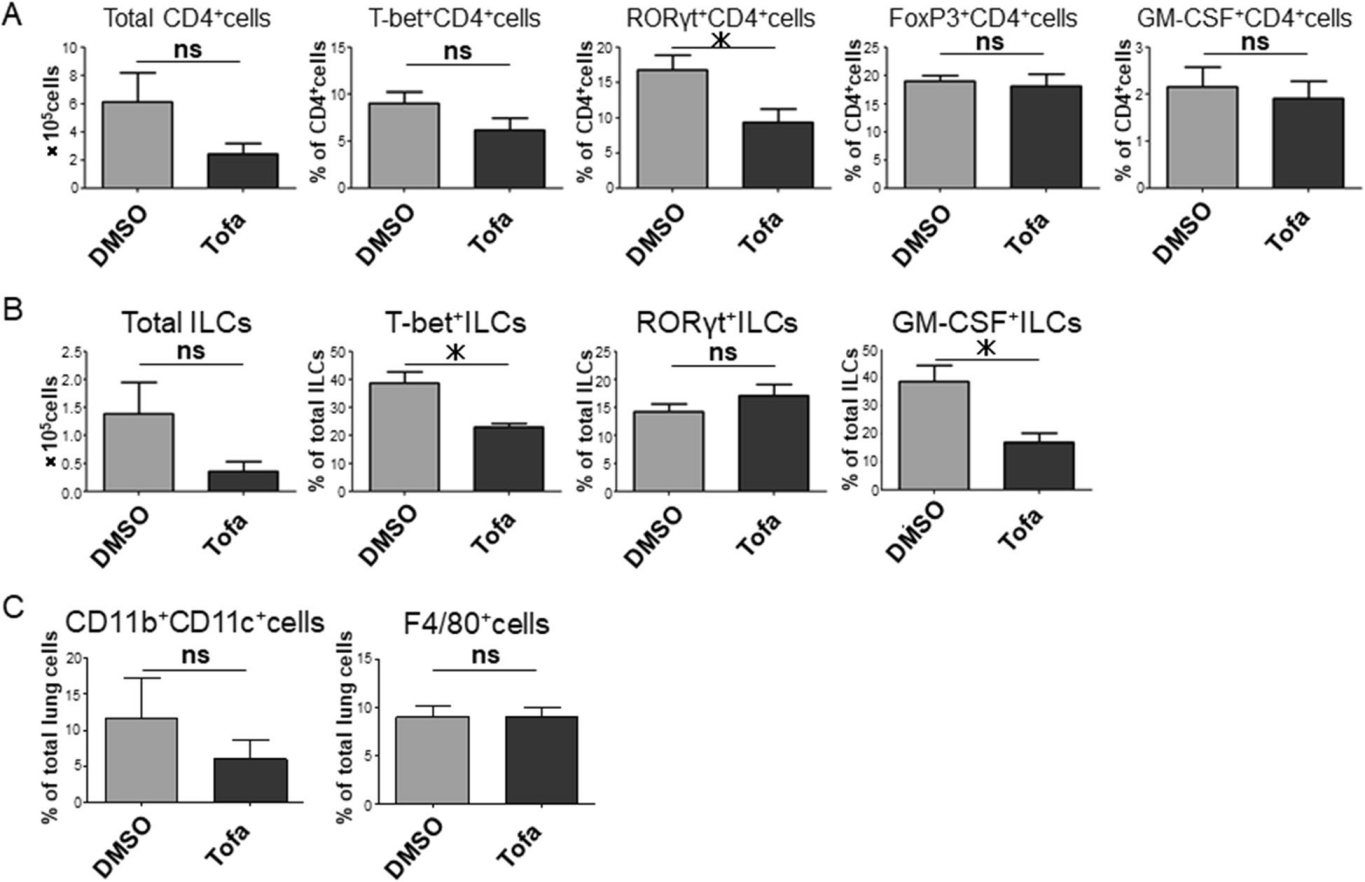
Tofacitinib suppresses Th17 cells, ILC1s, and GM-CSF^+^ ILCs in the lung. **a** Frequencies of CD4^+^ T cell subsets in the lung of control mice (*n* = 5) and tofacitinib-treated mice (*n* = 5). **b** ILC subsets in the lung of control mice (*n* = 5) and tofacitinib-treated mice (*n* = 5). **c** DCs and macrophages in the lung of control mice (*n* = 5) and tofacitinib-treated mice (*n* = 5). Data are shown as the mean ± SEM. *P < 0.05, Mann-Whitney *U* tests

We next compared the lung-infiltrating lymphoid cells between the tofacitinib-treated and DMSO-treated mice and demonstrated that the proportion of RORγt^+^CD4^+^ cells was decreased in the lung of tofacitinib-treated mice (Fig. 2a). In vitro experiments confirmed that tofacitinib suppresses CD4^+^ T cell proliferation and Th17 cell differentiation (Additional file 1: Figure S1). We also revealed that the number of T-bet^+^ ILCs and GM-CSF^+^ ILCs was significantly decreased in tofacitinib-treated mice (Fig. 2b). We further analyzed the lung-infiltrating myeloid cells. Tofacitinib tended to decrease DCs (CD11b^+^CD11c^+^ cells) in the lungs, but the difference was not significant between the tofacitinib-treated and control mice. There was no significant difference in the number of macrophages (F4/80^+^ cells) in the lungs (Fig. 2c). These results indicated that tofacitinib suppresses Zym-induced ILD by decreasing the expansion of Th17 cells, ILC1s, and GM-CSF-producing ILCs in SKG mice.

### Lung MDSCs are increased in the lungs of Tofacitinib-treated mice and suppress T cell proliferation and Th17 cell differentiation ex vivo

We next analyzed the lung-infiltrating myeloid cells. As we previously reported, the MDSCs were increased in the lungs of ILD-induced mice (DMSO).

**Fig. 3:**
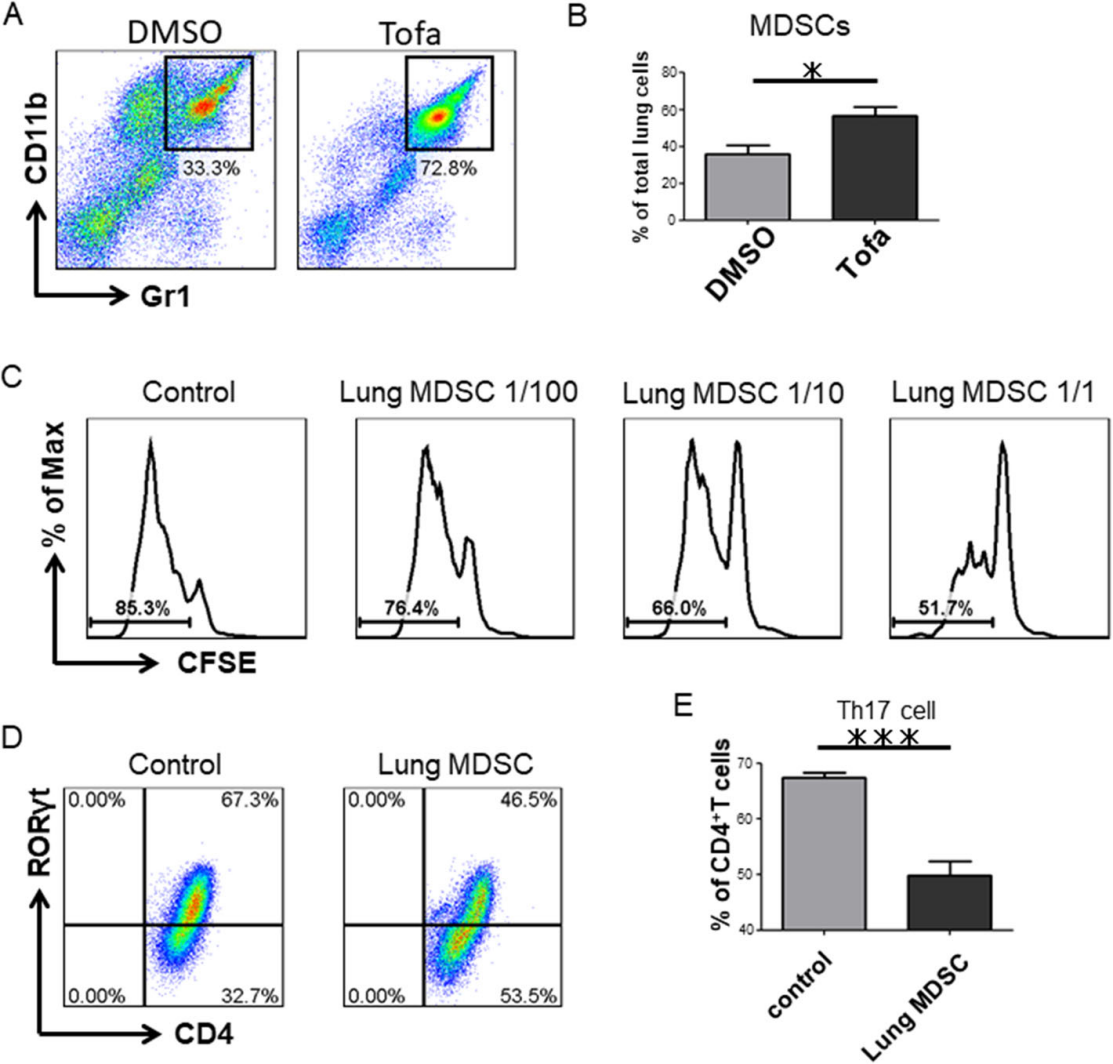
Lung MDSCs are increased in the lungs of tofacitinib-treated mice and suppress T cell proliferation and Th17 cell differentiation ex vivo. **a** Representative flow cytometry plots of MDSCs from the lungs of control mice and tofacitinib-treated mice. **b** Comparison of the lung-infiltrating MDSCs in control mice (*n* = 5) versus tofacitinib-treated mice (*n* = 5). **c** CFSE-labeled CD4^+^ T cells were cultured for 3 days with CD3 and CD28 stimulation, with or without the indicated proportion of lung MDSCs obtained from Zym-injected SKG mice. **d** Representative flow cytometry plots of the Th17 cell differentiation assay. In addition to the proliferation conditions, the following cytokines and antibodies were added: 10 ng/ml IL-6, 0.5 ng/ml TGF-β, 2.5 μg/ml anti-IFNγ, and 2.5 μg/ml anti-IL-4. **e** Proportion of Th17 cells obtained after CD4^+^ T cells were cultured with or without lung MDSCs (MDSCs, *n* = 5; control, *n* = 5). Data are shown as the mean ± SEM. *P < 0.05, ***P < 0.001, Mann-Whitney *U* tests

Interestingly, tofacitinib further increased the proportion of lung MDSCs (Fig. 3a, b). On the other hand, tofacitinib did not increase the CD11b^+^Gr1^dim^ tolDC-like cells. We then studied the effect of the isolated lung MDSCs on T cell proliferation and Th17 cell differentiation ex vivo. Lung MDSCs suppressed the proliferation of CD4^+^ T cells in an MDSC density-dependent manner (Fig. 3c). In addition, lung MDSCs suppressed the differentiation of CD4^+^ T cells into Th17 cells (Fig. 3d, e). These results indicated that tofacitinib has the potential to increase MDSCs in the lung and that the lung MDSCs can act to suppress pathogenic Th17 cells in the inflamed lung.

### Tofacitinib suppressed phosphorylation of STAT1 and STAT5, but not STAT3 in MDSCs

**Fig. 4:**
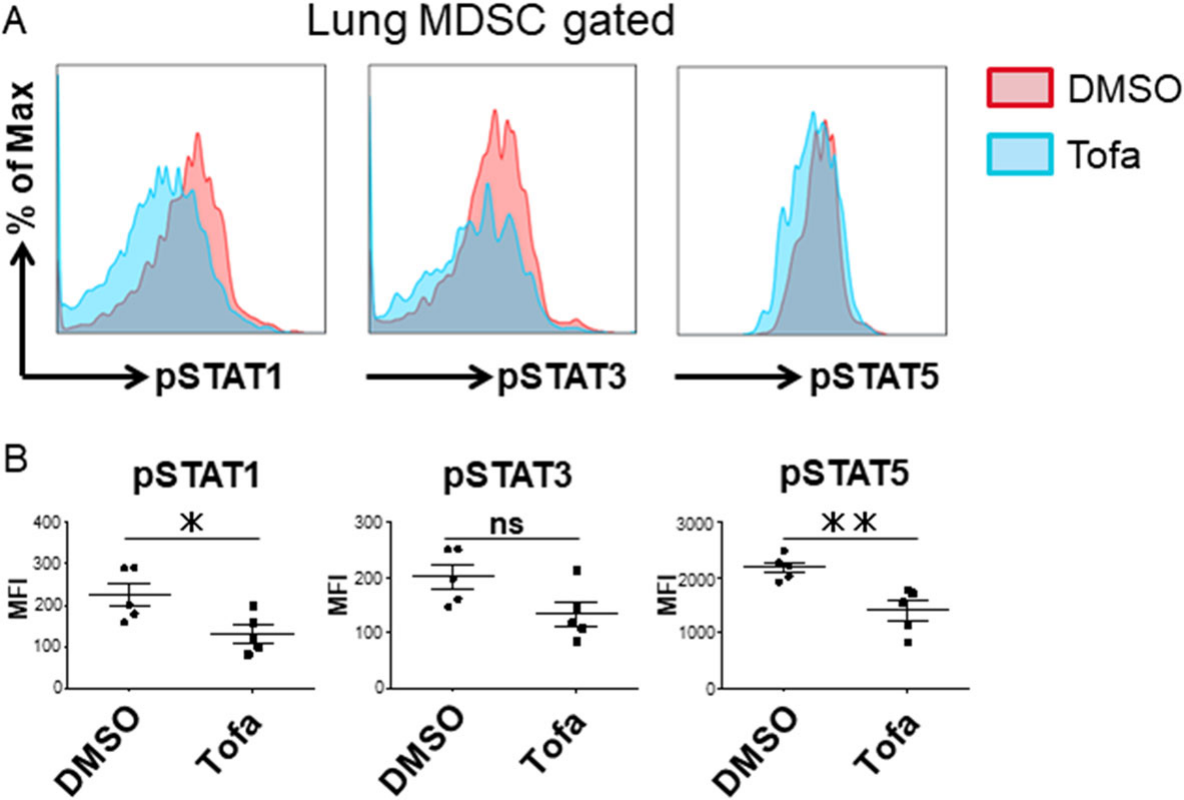
Tofacitinib inhibits the phosphorylation of STAT1 and STAT5 in MDSCs from the inflamed lung. **a** Representative flow cytometry plots of phosphorylated STATs in MDSCs from the lungs of control mice and tofacitinib-treated mice. **b** Comparison of mean fluorescence intensity (MFI) of phosphorylated STATs in lung-infiltrating MDSCs from control mice (*n* = 5) versus tofacitinib-treated mice (*n* = 5). Data are shown as the mean ± SEM. *P < 0.05, **P < 0.01, Student’s *t* tests

We next studied JAK-related proteins in MDSCs isolated from inflamed lungs. We revealed that the phosphorylation of STAT1 (pSTAT1) and STAT5 (pSTAT5) was significantly decreased in MDSCs from tofacitinib-treated mice compared to those from control mice. On the other hand, the phosphorylation of STAT3 (pSTAT3) was not suppressed in MDSCs from tofacitinib-treated mice (Fig. 4a, b).

### Tofacitinib suppresses DC differentiation and facilitates the expansion of MDSCs in vitro

We finally examined if tofacitinib increases the proportion of MDSCs in vitro.

**Fig. 5:**
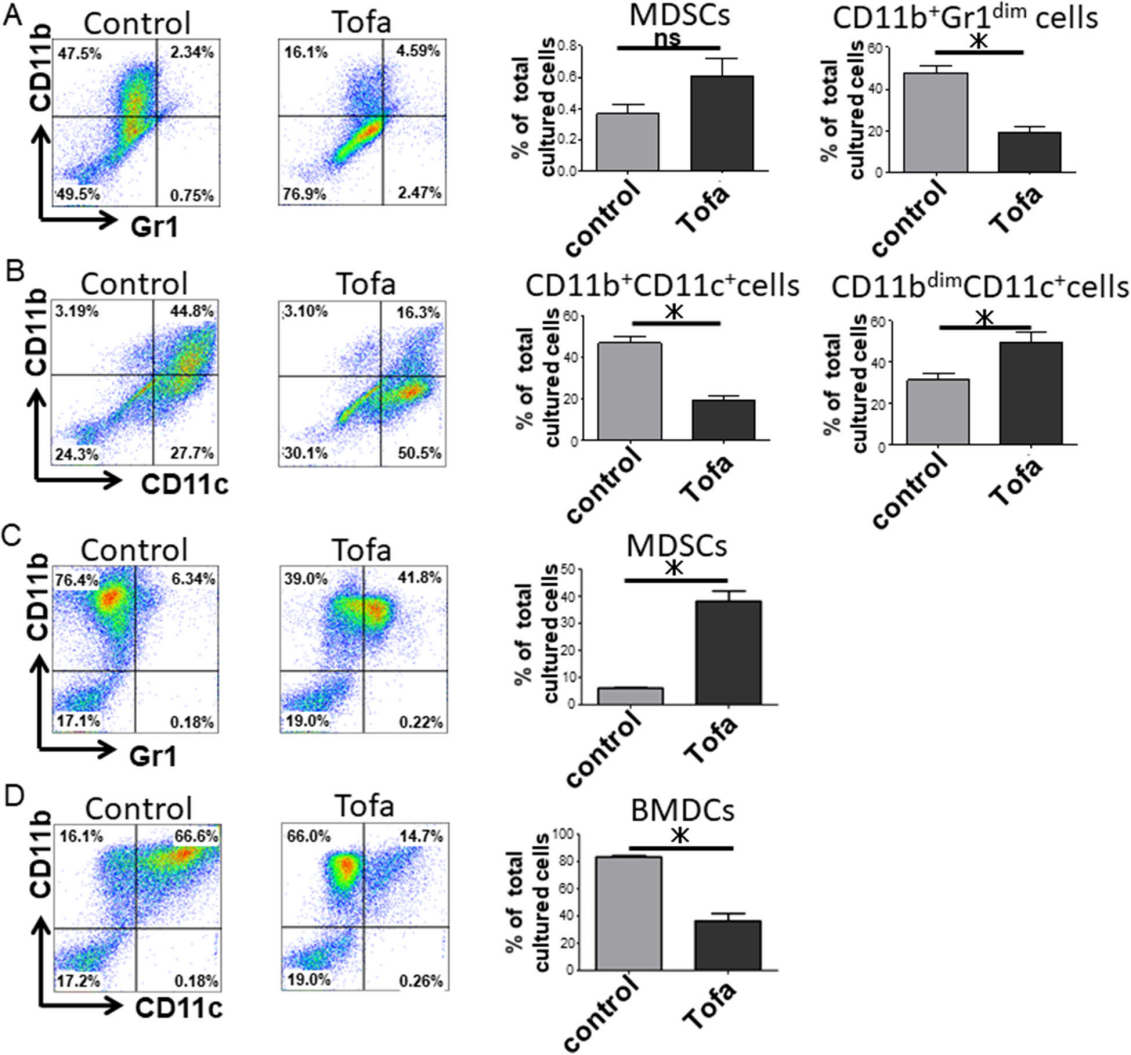
Tofacitinib facilitates the expansion of MDSCs in vitro. Lung-infiltrating cells (1 × 10^6^) (**a**, **b**) and BM cells (1 × 10^6^) (**c**, **d**) from naïve SKG mice were cultured with 100 ng/ml GM-CSF and 2.5 ng/ml rIL-4 for 5 days with or without tofacitinib. **a** Representative flow cytometry plots and the comparison of MDSCs and CD11b^+^Gr1^dim^ cells differentiated from lung-infiltrating cells cultured with or without tofacitinib (tofacitinib, *n* = 5; control, *n* = 5). **b** Representative flow cytometry plots and the comparison of CD11b^+^CD11c^+^ cells and CD11b^dim^CD11c^+^ cells differentiated from lung-infiltrating cells cultured with or without tofacitinib. **c** Representative flow cytometry plots and the comparison of MDSCs differentiated from BM cells cultured with or without tofacitinib (tofacitinib, *n* = 5; control, *n* = 5). **d** Representative flow cytometry plots and the comparison of BMDCs differentiated from BM cells cultured with or without tofacitinib. Data are shown as the mean ± SEM. *P < 0.05, Student’s *t* tests

Since MDSCs were increased in the lungs in vivo, we cultured the total lung-infiltrating cells under DC differentiation conditions with or without tofacitinib. Tofacitinib suppressed the differentiation of lung cells into CD11b^+^Gr1^dim^ cells (Fig. 5a), while it increased the proportion of CD11b^dim^CD11c^+^ cells (Fig. 5b). Flow cytometry showed very low MDSC distributions in both the tofacitinib and control groups (Fig. 5a). We next cultured BM cells under the same conditions with or without tofacitinib. Tofacitinib suppressed the differentiation of BM cells into BM-derived dendritic cells (BMDCs) and increased the proportion of MDSCs in vitro (Fig. 5c, d). These results indicated that tofacitinib expands MDSCs in the BM but not in the lung and that the expanded MDSCs may migrate into the inflamed lung.

## Discussion

In this study, we showed that tofacitinib suppresses the progression of ILD by facilitating the expansion of MDSCs in the lungs of SKG mice. The lung MDSCs suppressed CD4^+^ T cell proliferation and Th17 cell differentiation.

To our knowledge, this is the first report showing that tofacitinib is effective for treating the RA-ILD mouse model. Both CD4^+^ T cells and DCs contribute to the pathogenesis of RA-ILD [15–17]. Previous reports demonstrated that tofacitinib suppresses Th17 cell differentiation and DC activation [18–20]. We also demonstrated that tofacitinib suppresses CD4^+^ T cell proliferation, Th17 cell differentiation, and BMDC differentiation in vitro. Direct suppression of these pathogenic immune cells would be one of the reasons the tofacitinb suppresses the progression of ILD. In addition, we showed, in this study, that tofacitinib suppresses ILD through MDSC expansion.

We previously reported that tofacitinib facilitates the expansion of MDSCs in the BM and in the spleen and that it ameliorates arthritis in SKG mice [10], but we did not detect the expansion of MDSCs in the inflammatory site (e.g., joints). Importantly, in this study, we showed that tofacitinib increased the MDSCs in the inflamed lungs. We previously reported that MDSCs were increased in the early stages of ILD and that CD11b^+^Gr1^dim^ tolDC-like cells were increased in the late stages of ILD in SKG mice. Although we showed that CD11b^+^Gr1^dim^ tolDC-like cells inhibited the progression of ILD in SKG mice, the role of MDSCs in the inflamed lung was unclear. In this study, we showed that tofacitinib increased MDSCs but not CD11b^+^Gr1^dim^ tolDC-like cells in the lung. Furthermore, tofacitinib increased the proportion of MDSCs differentiated from BM cells but not from lung cells in vitro. These results indicated that MDSCs expand in the BM and migrate into the inflamed lungs, where they reduce lung inflammation in the early stage of ILD (Additional file 2: Figure S2). Consequently, there is no need to expand the CD11b^+^Gr1^dim^ tolDC-like cells.

It is known that GM-CSF is one of the important cytokines for the expansion of MDSCs. On the other hand, the combination of GM-CSF and IL-4 inhibits MDSC expansion by inducing their differentiation into mature DCs. The GM-CSF receptor transmits GM-CSF signaling associated with homodimer of JAK2, while the IL-4 receptor does IL-4 signaling with JAK1 and JAK3. We previously demonstrated that the number of MDSCs was increased by the addition of a selective JAK1 inhibitor or a selective JAK 3 inhibitor in vitro [10]. In contrast, a selective JAK 2 inhibitor did not affect the number of MDSCs. These results indicate that JAK2, but not JAK1 nor JAK3, signal is critical for the expansion of MDSCs. As tofacitinib inhibits signaling through JAK1 and JAK3 with 5–100-fold selectivity over JAK2 in cellular assays [21], GM-CSF signaling may transmit into the cells, while IL-4 signaling does not, resulting in MDSC expansion but not DC differentiation. In vivo concentration of tofacitinib might be above the inhibition levels of JAK1 and JAK3, but not that of JAK2, which facilitated the expansion of MDSCs in the lung of ILD-induced SKG mice.

It is known that STAT3 plays a critical role in the expansion of MDSCs [22]. Here, we showed that tofacitinib suppressed the phosphorylation of STAT1 and STAT5, but not STAT3 in MDSC from inflamed lungs. Thus, relatively higher STAT3 signaling might induce the MDSC expansion in the lungs.

## Conclusion

In summary, we demonstrated that tofacitinib suppresses the ILD in Zym-treated SKG mice. Although further studies are needed to elucidate the mechanism by which tofacitinib expands MDSCs, our study suggests that tofacitinib has a potential therapeutic effect for RA-ILD.

## Additional files

Additional file 1: **Figure S1.** Tofacitinib suppresses T cell proliferation and Th17 cell differentiation in vitro. a CFSE-labeled CD4^+^ T cells were cultured for 3 days with CD3 and CD28 stimulation, with or without the indicated concentration of tofacitinib. b Representative flow cytometry plots of the Th17 cell differentiation assay. In addition to the proliferation conditions, the following cytokines and antibodies were added: 10 ng/ml IL-6, 0.5 ng/ml TGF-β, 2.5 μg/ml anti-IFNγ, and 2.5 μg/ml anti-IL-4. c Proportion of Th17 cells obtained after CD4^+^ T cells were cultured with or without tofacitinib (1000 nM). Data are shown as the mean ± SEM. ***P < 0.001, Mann-Whitney *U* tests. (TIF 130 kb)

Additional file 2: **Figure S2.** Schematic summary of this study. Tofacitinib facilitates the expansion of MDSCs in BM, and the MDSCs migrate to the inflamed lungs. Expanded MDSCs suppress the Th17 cells, which in turn suppresses the progression of ILD. (TIF 104 kb)

## Data Availability

Data sharing is not applicable to this article as no datasets were generated or analyzed during the current study.

## Abbreviations

BM: Bone marrow
BMDCs: BM-derived dendritic cells
CFSE: Carboxyfluorescein diacetate succinimidyl ester
DCs: Dendritic cells
DMSO: Dimethyl sulfoxide
ILCs: Innate lymphoid cells
JAK: Janus kinase
MDA5: Melanoma differentiation-associated 5 gene
MDSCs: Myeloid-derived suppressor cells
MFI: Median fluorescence intensity
RA-ILD: Rheumatoid arthritis-associated interstitial lung disease
STAT: Signal transducer and activator of transcription
Tofa: Tofacitinib
Zym: Zymosan

## References

[1] Ota Mineto, Iwasaki Yukiko, Harada Hiroaki, Sasaki Oh, Nagafuchi Yasuo, Nakachi Shinichiro, Sumitomo Shuji, Shoda Hirofumi, Tohma Shigeto, Fujio Keishi, Yamamoto Kazuhiko (2016). Efficacy of intensive immunosuppression in exacerbated rheumatoid arthritis-associated interstitial lung disease. Modern Rheumatology.

[2] Tanaka Y, Maeshima K, Yamaoka K (2012). In vitro and in vivo analysis of JAK inhibitor in rheumatoid arthritis. Ann Rheum Dis.

[3] Kurasawa Kazuhiro, Arai Satoko, Namiki Yumeko, Tanaka Ayae, Takamura Yuta, Owada Takayoshi, Arima Masafumi, Maezawa Reika (2018). Tofacitinib for refractory interstitial lung diseases in anti-melanoma differentiation-associated 5 gene antibody-positive dermatomyositis. Rheumatology.

[4] Bronte V, Brandau S, Chen SH, Colombo MP, Frey AB, Greten TF (2016). Recommendations for myeloid-derived suppressor cell nomenclature and characterization standards. Nat Commun.

[5] Sendo S, Saegusa J, Morinobu A (2018). Myeloid-derived suppressor cells in non-neoplastic inflamed organs. Inflamm Regen.

[6] Sakaguchi N, Takahashi T, Hata H, Nomura T, Tagami T, Yamazaki S (2003). Altered thymic T-cell selection due to a mutation of the ZAP-70 gene causes autoimmune arthritis in mice. Nature.

[7] Sakaguchi S, Sakaguchi N, Yoshitomi H, Hata H, Takahashi T, Nomura T (2006). Spontaneous development of autoimmune arthritis due to genetic anomaly of T cell signal transduction: part 1. Semin Immunol.

[8] Shiomi A, Usui T, Ishikawa Y, Shimizu M, Murakami K, Mimori T (2014). GM-CSF but not IL-17 is critical for the development of severe interstitial lung disease in SKG mice. J Immunol.

[9] Sendo S, Saegusa J, Okano T, Takahashi S, Akashi K, Morinobu A (2017). CD11b+Gr-1^dim^ tolerogenic dendritic cell-like cells are expanded in interstitial lung disease in SKG mice. Arthritis Rheumatol.

[10] Nishimura K, Saegusa J, Matsuki F, Akashi K, Kageyama G, Morinobu A (2015). Tofacitinib facilitates the expansion of myeloid-derived suppressor cells and ameliorates arthritis in SKG mice. Arthritis Rheumatol.

[11] Yoshitomi H, Sakaguchi N, Kobayashi K, Brown GD, Tagami T, Sakihama T (2005). A role for fungal {beta}-glucans and their receptor Dectin-1 in the induction of autoimmune arthritis in genetically susceptible mice. J Exp Med.

[12] Nishikomori R, Ehrhardt RO, Strober W (2000). T helper type 2 cell differentiation occurs in the presence of interleukin 12 receptor beta2 chain expression and signaling. J Exp Med.

[13] Inaba K, Inaba M, Romani N, Aya H, Deguchi M, Ikehara S (1992). Generation of large numbers of dendritic cells from mouse bone marrow cultures supplemented with granulocyte/macrophage colony-stimulating factor. J Exp Med.

[14] Yoshida H, Kimura A, Fukaya T, Sekiya T, Morita R, Shichita T (2012). Low dose CP-690,550 (tofacitinib), a pan-JAK inhibitor, accelerates the onset of experimental autoimmune encephalomyelitis by potentiating Th17 differentiation. Biochem Biophys Res Commun.

[15] Turesson C, Matteson EL, Colby TV, Vuk-Pavlovic Z, Vassallo R, Weyand CM (2005). Increased CD4+ T cell infiltrates in rheumatoid arthritis-associated interstitial pneumonitis compared with idiopathic interstitial pneumonitis. Arthritis Rheum.

[16] Shimizu Y, Kuwabara H, Ono A, Higuchi S, Hisada T, Dobashi K (2006). Intracellular Th1/Th2 balance of pulmonary CD4(+) T cells in patients with active interstitial pneumonia evaluated by serum KL-6. Immunopharmacol Immunotoxicol.

[17] Yoshinouchi T, Ohtsuki Y, Ueda R, Sato S, Ueda N (1999). Myofibroblasts and S-100 protein positive cells in idiopathic pulmonary fibrosis and rheumatoid arthritis-associated interstitial pneumonia. Eur Respir J.

[18] Maeshima K, Yamaoka K, Kubo S, Nakano K, Iwata S, Saito K (2012). The JAK inhibitor tofacitinib regulates synovitis through inhibition of interferon-γ and interleukin-17 production by human CD4+ T cells. Arthritis Rheum.

[19] Kubo S, Yamaoka K, Kondo M, Yamagata K, Zhao J, Iwata S (2014). The JAK inhibitor, tofacitinib, reduces the T cell stimulatory capacity of human monocyte-derived dendritic cells. Ann Rheum Dis.

[20] Boor PPC, de Ruiter PE, Asmawidjaja PS, Lubberts E, van der Laan LJW, Kwekkeboom J (2017). JAK-inhibitor tofacitinib suppresses interferon alfa production by plasmacytoid dendritic cells and inhibits arthrogenic and antiviral effects of interferon alfa. Transl Res.

[21] Meyer DM, Jesson MI, Li X, Elrick MM, Funckes-Shippy CL, Warner JD (2010). Anti-inflammatory activity and neutrophil reductions mediated by the JAK1/JAK3 inhibitor, CP-690,550, in rat adjuvant-induced arthritis. J Inflamm (Lond).

[22] Condamine T, Gabrilovich DI (2011). Molecular mechanisms regulating myeloid-derived suppressor cell differentiation and function. Trends Immunol.

