# Supplementary figures and images for: Tofacitinib facilitates the expansion of myeloid-derived suppressor cells and ameliorates interstitial lung disease in SKG mice
Source: Arthritis Res Ther. 2019 Aug 6;21:184. doi: 10.1186/s13075-019-1963-2 (PMC6685227; doi:10.1186/s13075-019-1963-2)

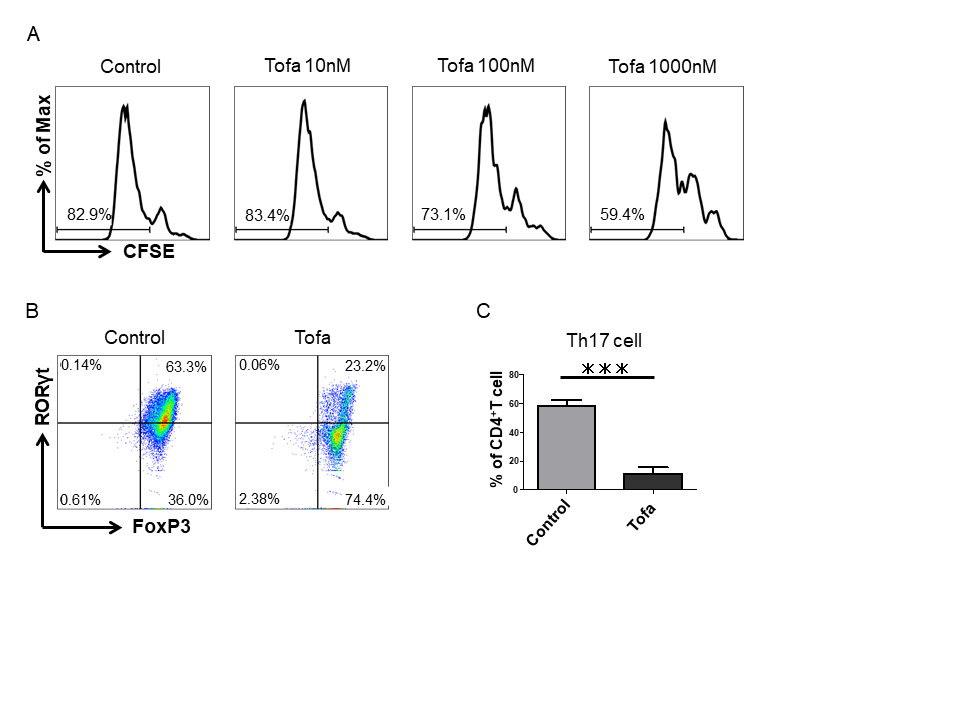

Supplement: Supplementary file 1 — Figure S1. Tofacitinib suppresses T cell proliferation and Th17 cell differentiation in vitro. a CFSE-labeled CD4+ T cells were cultured for 3 days with CD3 and CD28 stimulation, with or without the indicated concentration of tofacitinib. b Representative flow cytometry plots of the Th17 cell differentiation assay. In addition to the proliferation conditions, the following cytokines and antibodies were added: 10 ng/ml IL-6, 0.5 ng/ml TGF-β, 2.5 μg/ml anti-IFNγ, and 2.5 μg/ml anti-IL-4. c Proportion of Th17 cells obtained after CD4+ T cells were cultured with or without tofacitinib (1000 nM). Data are shown as the mean ± SEM. ***P < 0.001, Mann-Whitney U tests. (TIF 130 kb) [file 13075_2019_1963_MOESM1_ESM.tif]

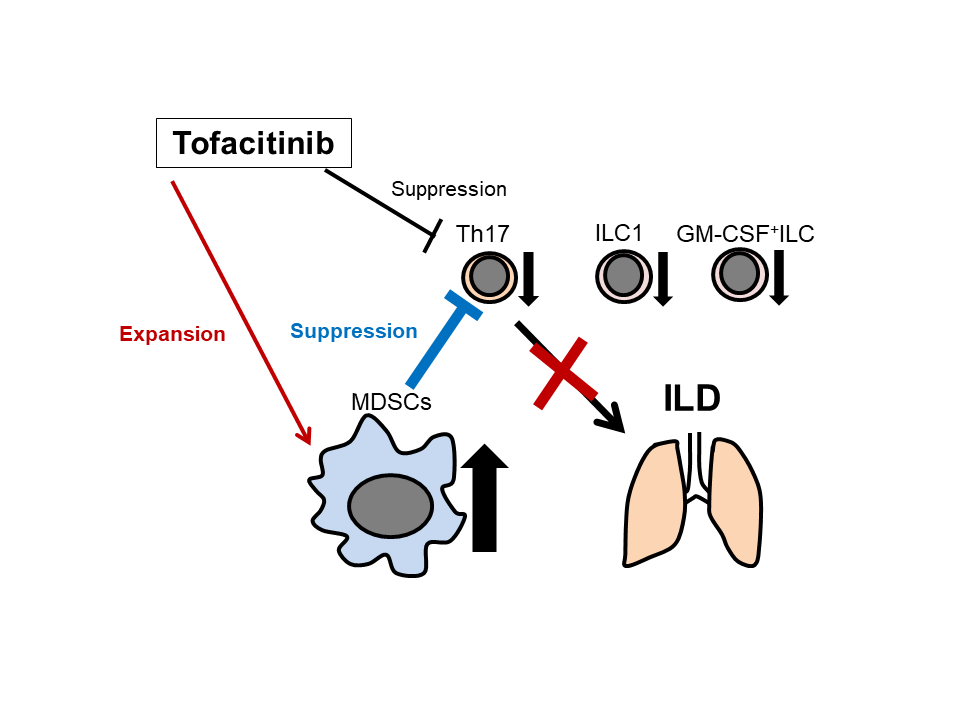

Supplement: Supplementary file 2 — Figure S2. Schematic summary of this study. Tofacitinib facilitates the expansion of MDSCs in BM, and the MDSCs migrate to the inflamed lungs. Expanded MDSCs suppress the Th17 cells, which in turn suppresses the progression of ILD. (TIF 104 kb) [file 13075_2019_1963_MOESM2_ESM.tif]
